# Fault detection by skeleton extraction based on orientation field consistency

**DOI:** 10.1371/journal.pone.0271615

**Published:** 2022-07-15

**Authors:** Yang Li, Baorong Zhong, Xiaohong Xu, Zijun Liang

**Affiliations:** 1 School of Geosciences, Yangtze University, Wuhan, Hubei, China; 2 School of Computer Science, Yangtze University, Jingzhou, Hubei, China; 3 Data Company of the Xinjiang Oilfield Company of PetroChina, Karamay, Xinjiang, China; Universidade Federal de Uberlandia, BRAZIL

## Abstract

A fault detection method using skeleton extraction based on orientation field consistency is proposed to improve the efficiency of fault detection, reduce the influence of transverse nonstructural factors on fault detection, and realize automatic fault extraction. In fingerprint image processing, the consistency of the orientation field reaches a maximum value when all orientations are parallel and takes a smaller value when not all orientations are parallel. The orientation field ceases to be parallel in the presence of a stratigraphic discontinuity, and the consistency of the orientation field in the corresponding region is lower than that in parallel regions. This characteristic can be exploited to extract discontinuous regions from seismic data. Then, binarization and closing operations are used to extract fault areas and increase fault continuity. Finally, a skeleton extraction method based on extracting the longitudinal center point is used to identify the fault lines. Compared with the classical ant tracking method, the proposed method requires the adjustment of fewer parameters, thus simplifying fault identification process to a certain extent. Moreover, the proposed method effectively suppresses transverse discontinuities, highlights the longitudinal fault characteristics, and strengthens fault continuity.

## Introduction

Fault interpretation in seismic data is the basis of seismic data interpretation. A displacement or lack of a seismic reflection layer, discontinuities in the transverse amplitude, and significant differences in the reflection amplitude on either side of a fault are used to intuitively detect faults as the main means of manually drawing fault profiles. However, the visual detection efficiency of this fault detection method is low, its subjectivity is high, and the interpretability of the results is limited.

To improve the efficiency of fault identification, the coherence cube approach [1–4], chaos [5, 6], and curvature [7–9] can all be used for fault and fracture detection. The introduction of such algorithms weakens the transverse continuity of seismic data, enhances discontinuities, and greatly improves the efficiency of fault detection. However, these algorithms extract only fault enhancement values and require the fault lines to be drawn artificially.

For the automatic extraction of fault lines and planes, ant tracking [10] can be used to extract fault lines based on curvature [11, 12], chaos [13–15], and the coherence cube [16, 17]. However, many parameters need to be considered for the ant tracking method, and adjustments to these parameters substantially affect the accuracy of fault detection. In addition, improper parameter adjustment can cause nonstructural factors to have a major impact on the fault detection results.

With the ongoing development of artificial intelligence techniques, scholars have increasingly used artificial neural networks [18], deep neural networks [19], and convolutional neural networks [20–22] for fault detection. Although these methods can improve the accuracy of fault detection and realize intelligent detection, they depend on the availability of many fault samples and have a long training time. These approaches are essentially supervised classification and prediction methods for fault detection.

To better extract additional fault data under unsupervised conditions, draw fault lines more accurately and automatically, and eliminate the problem of excessive sensitivity to the ant tracking parameters, we propose a skeleton extraction method based on orientation field consistency for detecting faults from seismic data. Kass and Witkin (1987) [23] proposed the concept of consistency, which mainly refers to the measurement of the anisotropy intensity reflecting the local orientation field information for each orientation using fingerprint image edge detection techniques. The orientation field consistency is the largest when all orientations are parallel and the smallest when all orientations are not parallel. This feature can be used to extract discontinuous regions from seismic data. Next, the approximate contours of the extracted faults can be determined by applying morphological binarization and closing operations [24, 25], after which a skeleton extraction method is used to extract the fault lines. Common skeleton extraction methods, such as those of Zhang-Suen [26] and Lee [27], the Khalid–Marek–Mariusz–Marcin (K3M) method [28], and medial axis skeletonization [29, 30], tend to produce burrs because of their lack of control over the longitudinal extraction trend. Therefore, this paper proposes a new skeletonization method designed to reduce the burrs caused by nonstructural factors to realize the extraction of fault lines. Different from a fault detection method based on a neural network, the method proposed in this paper for extracting fault lines does not depend on samples and operates in an unsupervised manner. The proposed method is compared with the coherence cube, chaos, variance, and ant tracking methods to confirm its effectiveness and advantages.

## Materials

The study data used in this paper are seismic data in the SEG-Y format. The SEG-Y format is a standard tape data format proposed by the Society of Exploration Geophysicists (SEG), and it is one of the most common formats used for seismic data in the petroleum exploration industry. The finest level of granularity in SEG-Y is trace data. These traces form Xlines (XLs) and Inlines (ILs), which represent seismic waves in the X-direction and Y-direction, respectively. The discontinuity of seismic waves is the key to fault identification. In the SEG-Y format, seismic data are represented by spatial distances in the XL and IL directions and by time in the depth direction.

**Fig 1:**
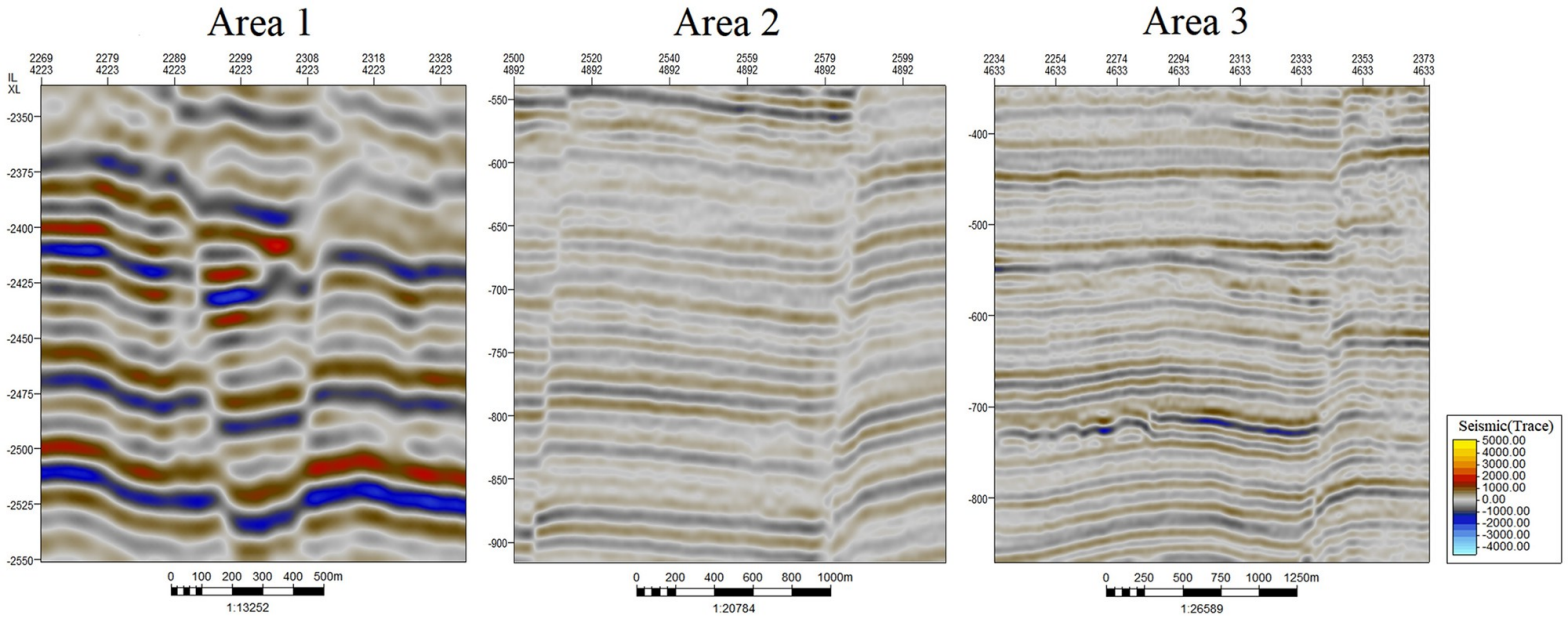
Test SEG-Y seismic data of different scales used in this paper.

The seismic data used in this paper are from Parihaka in New Zealand (https://wiki.seg.org/). To verify the effectiveness of the proposed method, this paper uses three subsets of SEG-Y seismic data in different ranges as test data. In the seismic data used in this paper, the IL interval is 25 meters (m), the XL interval is 12.5 m, and the depth interval is 3 milliseconds (ms). The test SEG-Y seismic data profiles are intercepted in XLs. Therefore, faults are extracted on 2D planes in this paper. The smallest traces in the SEG-Y data have dimensions of 25 m*3 ms. We can construct the 3D fault lines by detecting faults on all 2D planes. Fig 1 shows three seismic profiles in different ranges:

1. The range of area 1 in Fig 1 is 1600 m*216 ms and 64*1*72, which means that the IL distance is 1600 m, the time extent in the depth direction is 216 ms, the number of traces in the depth direction is 72, the number of ILs is 64, and the number of XLs is 1. Accordingly, the number of traces is 64*72. In this paper, we consider area 1 as an image with a size of 64*72. The pixels of this image represent the trace values; this is also the case for areas 2 and 3. In area 1 in Fig 1, there are two geological faults, and the development orientation of the faults is relative, so geological grooves are produced.
2. The range of area 2 in Fig 1 is 2775 m*378 ms and 111*1*126, which means that the IL distance is 2775 m, the time extent in the depth direction is 378 ms, the number of traces in the depth direction is 126, the number of ILs is 111, and the number of XLs is 1. Accordingly, the number of traces is 111*126. In this paper, we consider area 2 as an image with a size of 111*126.
3. The range of area 3 in Fig 1 is 3550 m*525 ms and 142*1*175, which means that the IL distance is 3550 m, the time extent in the depth direction is 525 ms, the number of traces in the depth direction is 175, the number of ILs is 142, and the number of XLs is 1. Accordingly, the number of traces is 142*175. In this paper, we consider area 3 as an image with a size of 142*175.

## Methods

**Fig 2:**
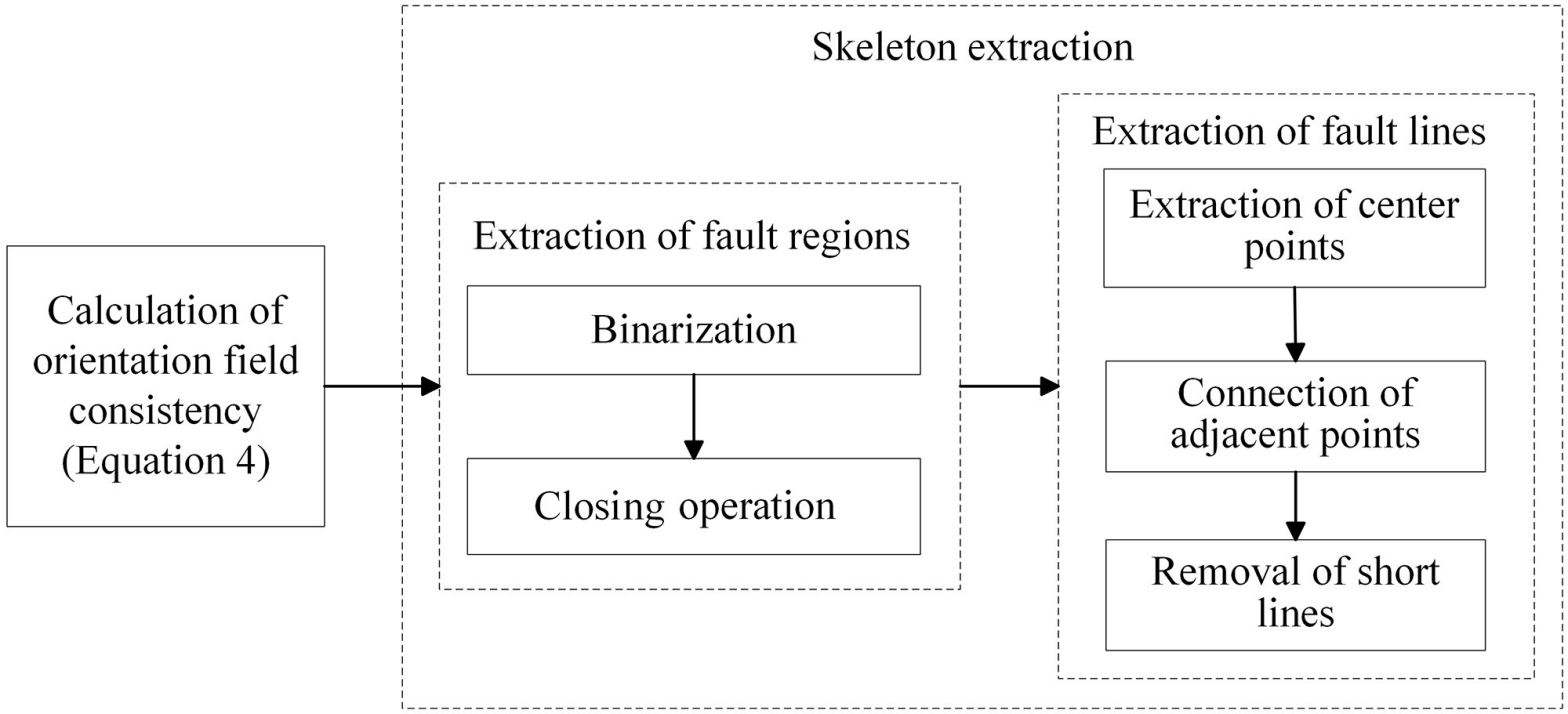
Flowchart of fault detection.

The proposed method for fault extraction from seismic data is presented below (Fig 2).

1. Longitudinal discontinuities in the seismic data are extracted by calculating the consistency of the orientation field.
2. Binarization and closing operations are used to suppress laterally discontinuous nonstructural factors of geological development.
3. Center point extraction, connection of adjacent points, and removal of short lines are performed to extract the skeleton in order to identify longitudinally discontinuous faults.

### Orientation field consistency

Kass and Witkin [23] proposed the concept of orientation field consistency, which is described here. The orientation at any point (x,y) in a grayscale image can be defined in terms of the angle between the tangent orientation of the ridge line (or valley line) at that point and the horizontal axis. The orientation field consistency reaches a maximum value when all orientations are parallel and a minimum value when all orientations are not parallel. These maximum and minimum values represent the extremes between which the consistency of the orientation field may vary. Accordingly, the orientation field consistency can be used to measure the degree of order of the orientation field. In seismic data analysis, a continuous seismic wave has a high orientation field consistency, whereas a discontinuous seismic wave has a low orientation field consistency. Thus, a fault-enhanced data volume can be extracted based on the orientation field consistency. The procedure for determining the orientation field consistency is described below.

For a given two-dimensional (2D) seismic data volume I (composed of traces) expressed in a Cartesian coordinate system, the gradient can be expressed as ∇*I* = [*I*_*x*_, *I*_*y*_]. First, the derivatives at each data point (x,y) in the X and Y directions are taken to obtain the corresponding gradient vectors *G*_*xx*_ and *G*_*yy*_, respectively. In the present study, these gradients are calculated using the Sobel operator [31]. Then, the squared gradient vectors are convolved using Eqs 1, 2, and 3, where W denotes the convolution kernel.

$$\begin{array}{c} G_{xx}=\sum_{W}I_{x}^{2} \end{array}$$ (1)

$$\begin{array}{c} G_{yy}=\sum_{W}I_{y}^{2} \end{array}$$ (2)

$$\begin{array}{c} G_{xy}=\sum_{W}I_{x}I_{y} \end{array}$$ (3)

The orientation field consistency as calculated in Ref. [18] is given below.

$$\begin{array}{c} Coh=\frac{\sqrt{{\left(G_{xx}-G_{yy}\right)}^{2}+4G_{xy}^{2}}}{G_{xx}+G_{yy}} \end{array}$$ (4)

A low orientation field consistency *Coh* in Eq 4 indicates low continuity of the corresponding seismic wave, possibly reflecting a discontinuous region such as a fault, a fragmented stratum, noise, or lithological changes, while a large *Coh* indicates high stratigraphic continuity, complete development, and low damage in the region of interest. Algorithm 1 below presents code based on OpenCV for determining the consistency of an orientation field.

**Algorithm 1** Algorithm based on OpenCV for determining the consistency of an orientation field

**Fig 3:**
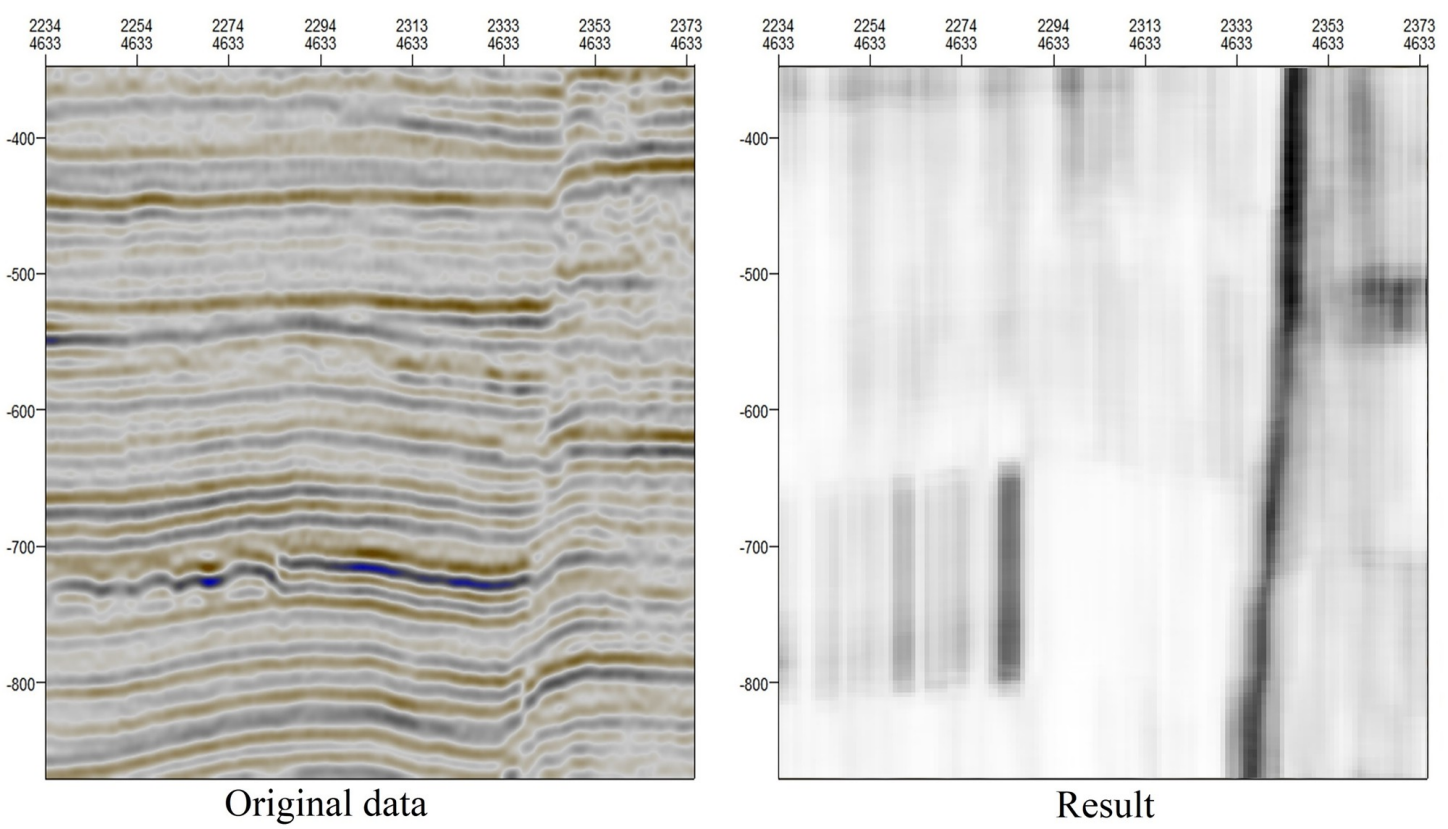
Fault enhancement result extracted based on orientation field consistency.

**Input:** The original data, as shown in Fig 3

**Output:** The consistency result, as shown in Fig 3

 *I*_*x*_ = *cv*2.*Sobel*(*Input*, *cv*2.*CV*_16*SC*1, 1, 0)

 *I*_*y*_ = *cv*2.*Sobel*(*Input*, *cv*2.*CV*_16*SC*1, 0, 1)

 *W* = *np*.*ones*(*m*, *n*)

 $G_{xx}=cv2.filter2D\left(I_{x}^{2},-1,W\right)$

 $G_{yy}=cv2.filter2D\left(I_{y}^{2},-1,W\right)$

 $G_{xy}=cv2.filter2D\left(I_{xy}^{2},-1,W\right)$

 $Output\left(Coh\right)=\frac{\sqrt{{\left(G_{xx}-G_{yy}\right)}^{2}+4G_{xy}^{2}}}{G_{xx}+G_{yy}}$

When extracting the fault enhancement based on the orientation field consistency, the parameter to be adjusted is the convolution kernel (W in algorithm 1). Different convolution kernels have different visual effects. Commonly used convolution kernels are generally square matrices, with dimensions of m*m. In contrast, this paper adopts a convolution kernel of *W* = m*n, that is, a matrix of m*n, in which all matrix elements are 1. When *m* > *n*, the extracted longitudinal features will be more obvious, and transverse discontinuities will be suppressed.

Fig 3 shows that the convolution results (*W* = 40*8). The values output by algorithm 1) are between 0 and 1. However, from the perspective of computer vision, there is little difference between 0 and 1 when mapped to the color space. Instead, the value range of grayscale images, on which many image processing technologies are based, is from 0 to 255. In computer vision, a value of 0 visually appears as black, and a value of 255 appears as white. Thus, we apply normalization to map the fault enhancement values in the range of 0 to 1 to values between 0 and 255.

The orientation field consistency represents the continuity of seismic waves in the transverse orientation and reflects the integrity of the stratum. When the local seismic waves are parallel, the consistency of the orientation field approaches 255, and the development of the stratum is relatively complete. When the seismic waves are not parallel, the consistency of the orientation field tends toward 0, and the stratum development may be broken or the lithology may be variable. Therefore, the darker the color is in the result image in Fig 3, indicating pixel values closer to 0, the smaller the orientation field consistency, indicating worse horizontal continuity of the seismic wave and a greater possibility of stratum discontinuity and faults. In contrast, the lighter the color is in the result image in Fig 3, indicating pixel values closer to 255, the greater the consistency of the orientation field, indicating stronger horizontal continuity of the seismic wave, better continuity of the stratum, and a lower likelihood of fault occurrence.

When the pixel values in a region are close to 0, this does not necessarily mean that there is a fault in this region. It also may be that the seismic wave is discontinuous due to changes in lithology, while the stratum may still be continuous. Such regions manifest in the form of small dark areas. These small dark areas have two characteristics: their distribution is scattered and has no obvious longitudinal component. They may interfere with fault detection. In contrast, dark areas containing faults have the following two characteristics: their distribution is concentrated and exhibits obvious longitudinal continuity. In the next subsection, in accordance with these characteristics of faults, we propose a new skeleton extraction method to detect faults in stratum continuity while reducing the interference of lithological changes on fault detection.

### Skeleton extraction

The extraction of the orientation field consistency yields only the fault enhancement values. Geologists often use a fault line to represent a fault on a 2D plane. A fault line is an abstract form of a fault. Therefore, the extracted binary fault area must be further vectorized to realize skeleton extraction in the fault area to obtain the fault lines, thus reducing the workload of manually drawing fault lines. To realize fault line extraction in a binary region while eliminating the disadvantage of excessive burrs in traditional skeleton extraction, this paper proposes a new skeleton extraction method.

#### Extraction of fault regions

As described above, we extract and normalize the consistency of the orientation field to obtain fault enhancement values between 0 and 255. For fault detection, we are only concerned with fault areas and nonfault areas. Therefore, we first use binarization to extract the fault areas. Through binarization, we can convert a grayscale fault enhancement image into a fault area image. Then, we exchanged black and white areas by color inversion.

**Fig 4:**
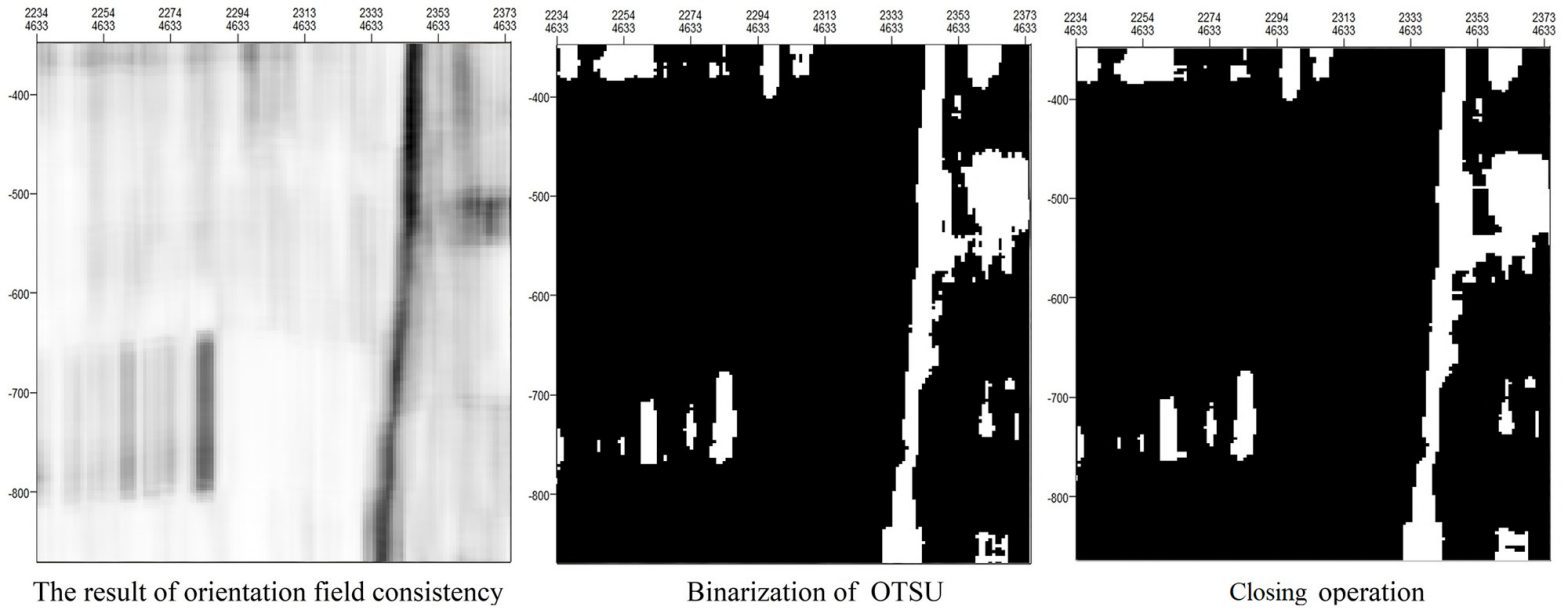
Fault areas extracted by means of the OTSU binarization method and the closing operation.

As shown in Fig 4, a value of 0 is depicted as black and represents a nonfault area, and a value of 255 is depicted as white and represents a fault area. When extracting fault areas through binarization, the threshold value used for the binarization of the grayscale image needs to be determined, and different parameter values will produce different results. In this paper, the binary method of OTSU [32] is used to automatically extract fault areas. Then, the closing operation is used to ensure the fault continuity. Fig 4 shows the results of OTSU binarization and the closing operation.

#### Extraction of center points

**Fig 5:**
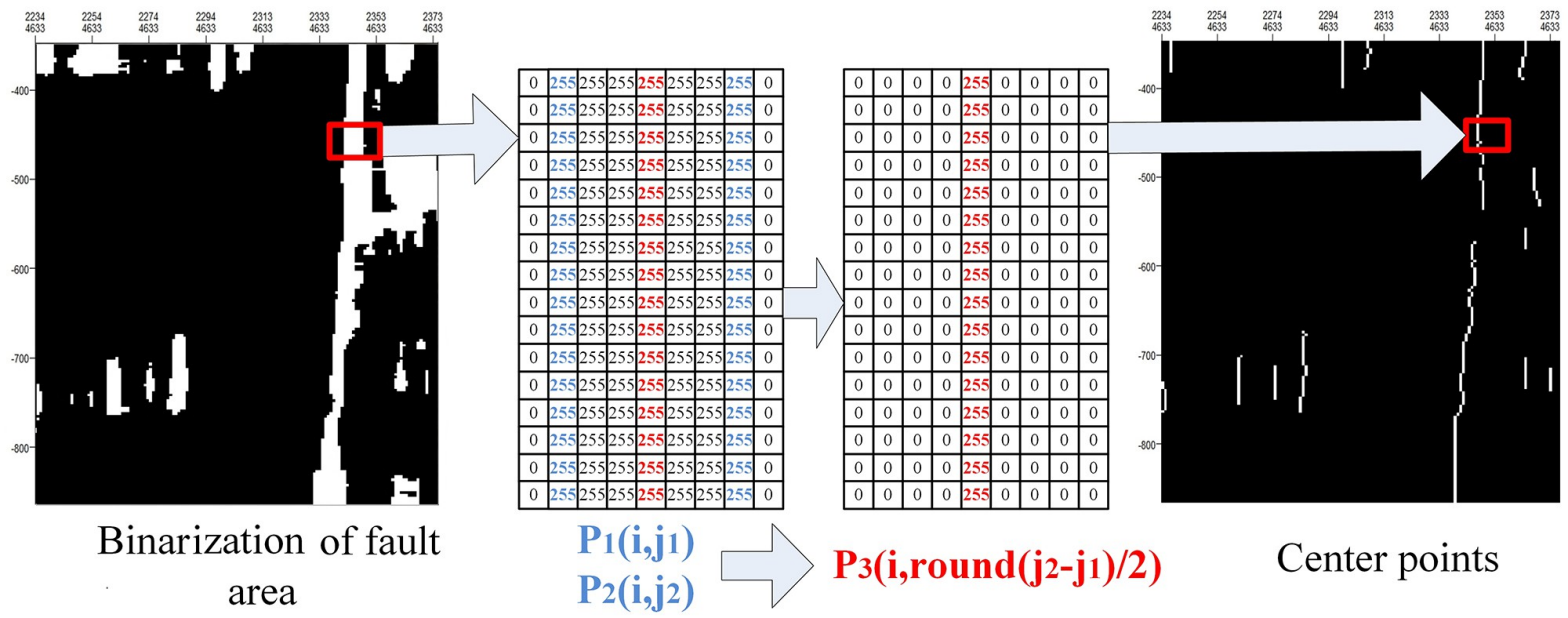
Extraction of center points.

Since fault development mainly occurs in the longitudinal direction, center points are extracted longitudinally. The extraction effect is shown in Fig 5. In Fig 5, white represents a value of 255, and black represents a value of 0. The specific steps are as follows:

1. Obtain the white areas after binarization.
2. Traverse each white area row by row to find the center point of each row. As shown in Fig 5, *P*_1_ and *P*_2_ represent the boundary of a row in the white area. *P*_3_ represents the center point between *P*_1_ and *P*_2_ in each row. *i* stands for row *i*, *j* stands for column *j*. *P*_1_(*i*, *j*_1_) represents the starting point of the white area in row *i*, and *P*_2_(*i*, *j*_2_) represents the end point of the white area in row *i*. Round indicates mathematical rounding. (*i*, *round*(*j*_2_−*j*_1_)/2) indicates the coordinates of the center point (*P*_3_) of the *i* row (red 255 in Fig 5).

#### Connection of adjacent points

**Fig 6:**
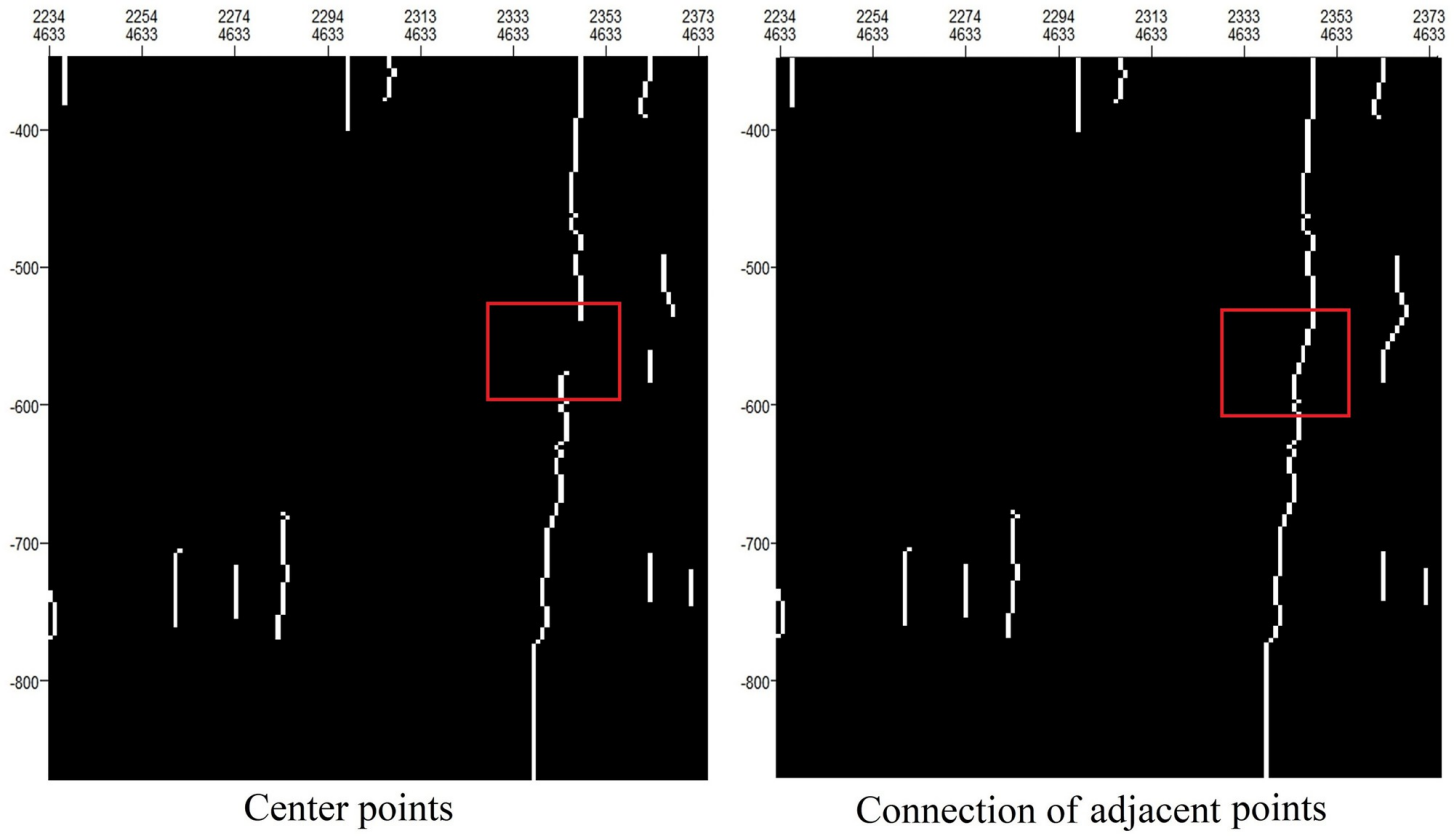
Connection of adjacent points.

To extract a fault line, its corresponding center points must be connected. In accordance with the principle of eight-neighborhood connectivity, some adjacent points can be regarded as line segments. By traversing all line segments, we can determine whether the distance between one of the two endpoints of a line segment and an endpoint of another line segment is less than D. If so, we connect those two endpoints. Fig 6 shows the fault line obtained with D = 15 for the considered example.

#### Removal of short lines

**Fig 7:**
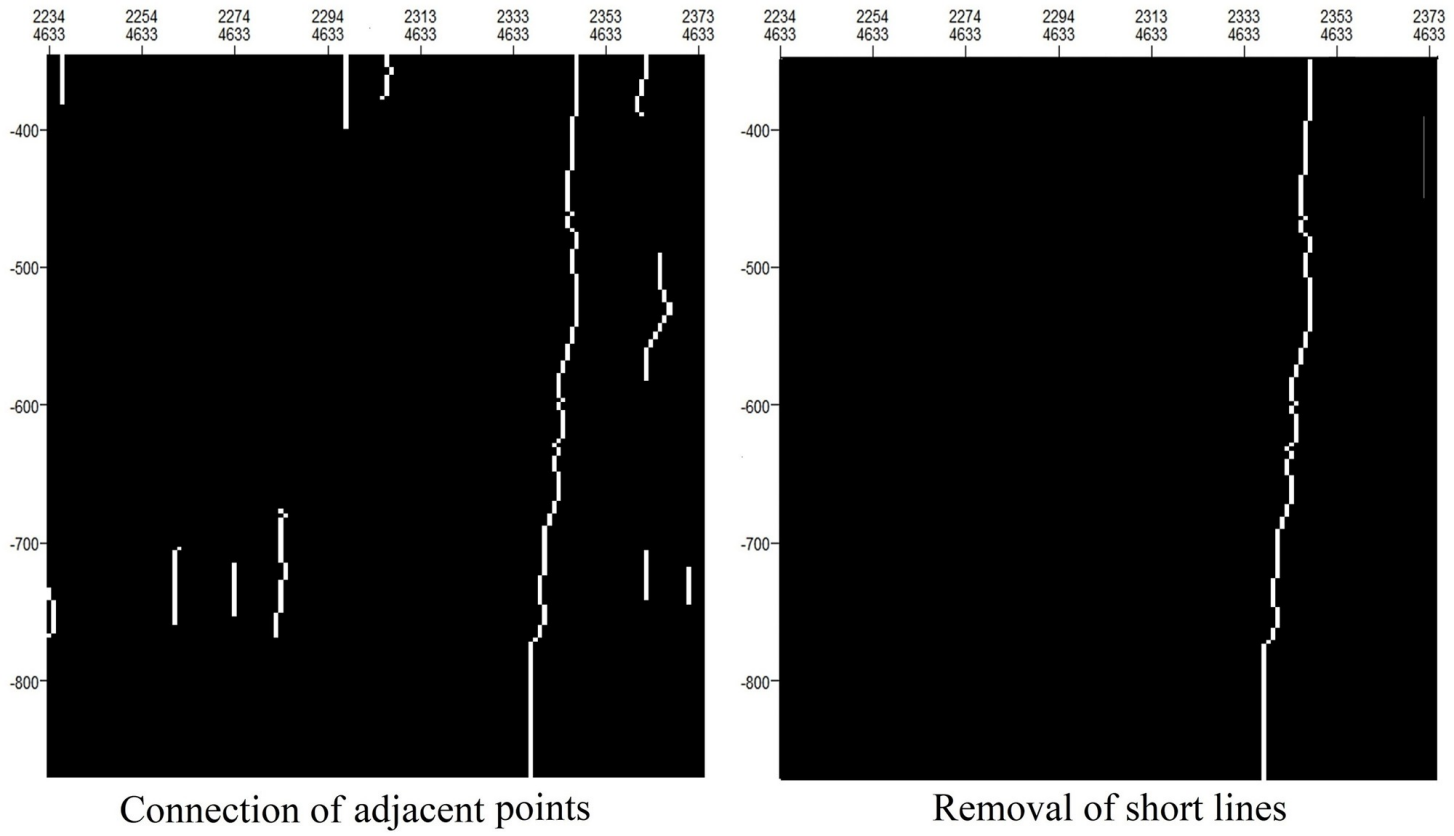
Removal of short lines.

The skeleton extraction method described above may produce anomalies. For example, discontinuities in seismic waves caused by lateral nonstructural factors will also be reflected as longitudinal fault lines. Therefore, excessively short line segments need to be removed. We remove the current line segment when its length is shorter than a certain threshold length. The image after the connection of adjacent points in Fig 7 comes from Fig 6.

## Results and discussion

### Optimal parameter adjustment

The process of fault detection via skeleton extraction based on orientation field consistency is described above. For this process, the optimal parameters of the convolution kernel (W), the minimum distance between adjacent unconnected points (D), and the minimum length threshold for the removal of short line (length) are still unknown. Next, we will use three areas of different scales as examples to illustrate how to adjust the parameters to obtain the optimal fault extraction results.

**Fig 8:**
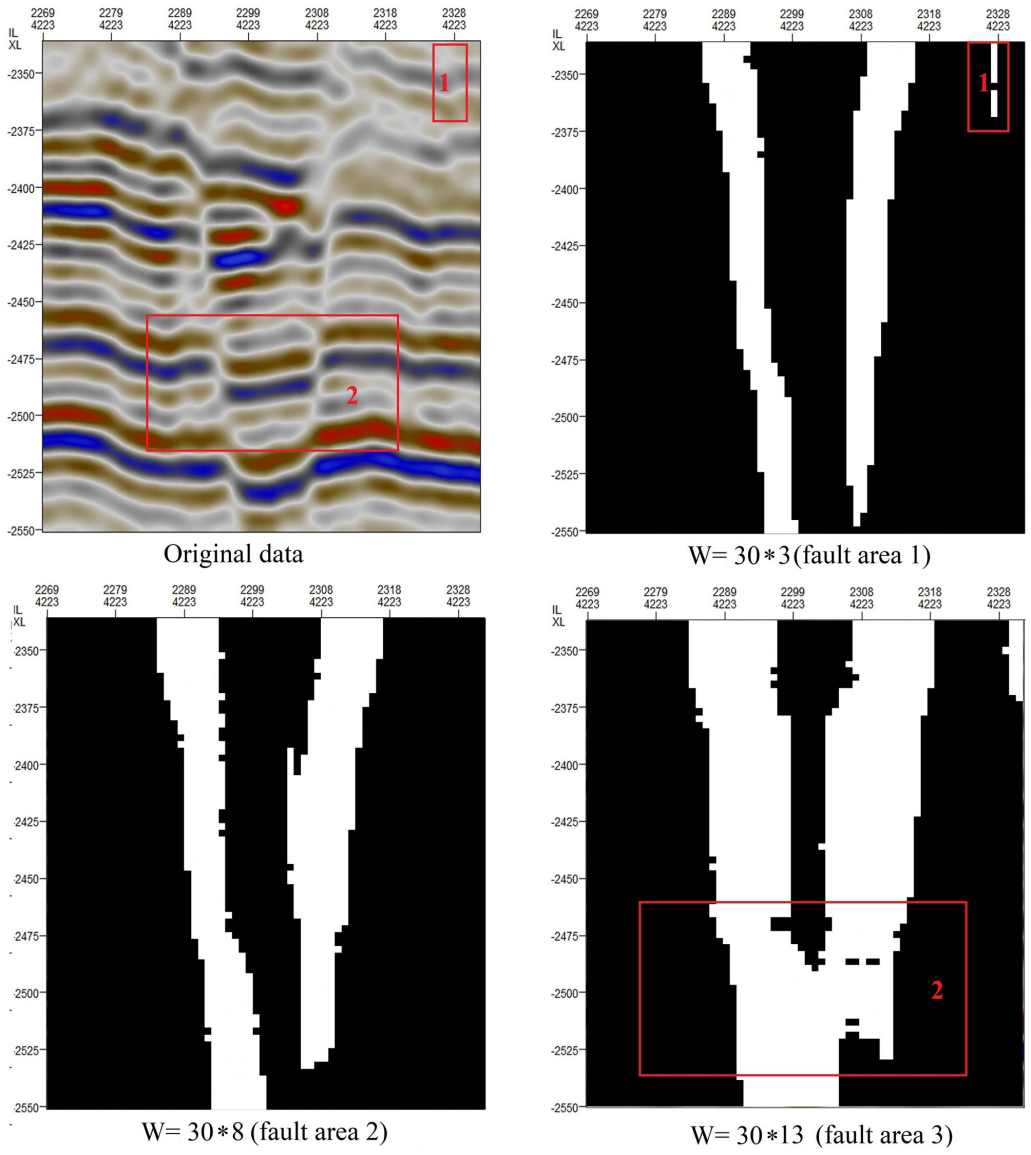
Influence of different values of n in the convolution kernel W on the extracted fault areas.

For the method introduced above, the convolution kernel used to extract the orientation field consistency is very important to the extraction of fault areas. For a convolution kernel of the form W = m*n, Fig 8 shows the effects of different values of n on fault extraction. We can draw the following conclusions: When n = 3, a false fault region is extracted (red-boxed region in fault area 1). Since the minimum distance between two faults is 10, when n = 13, two distinct faults are combined into one fault (red-boxed region in fault area 3). Through testing on many fault areas of different scales, we have found that the best range of n is between 5 and 8.

**Fig 9:**
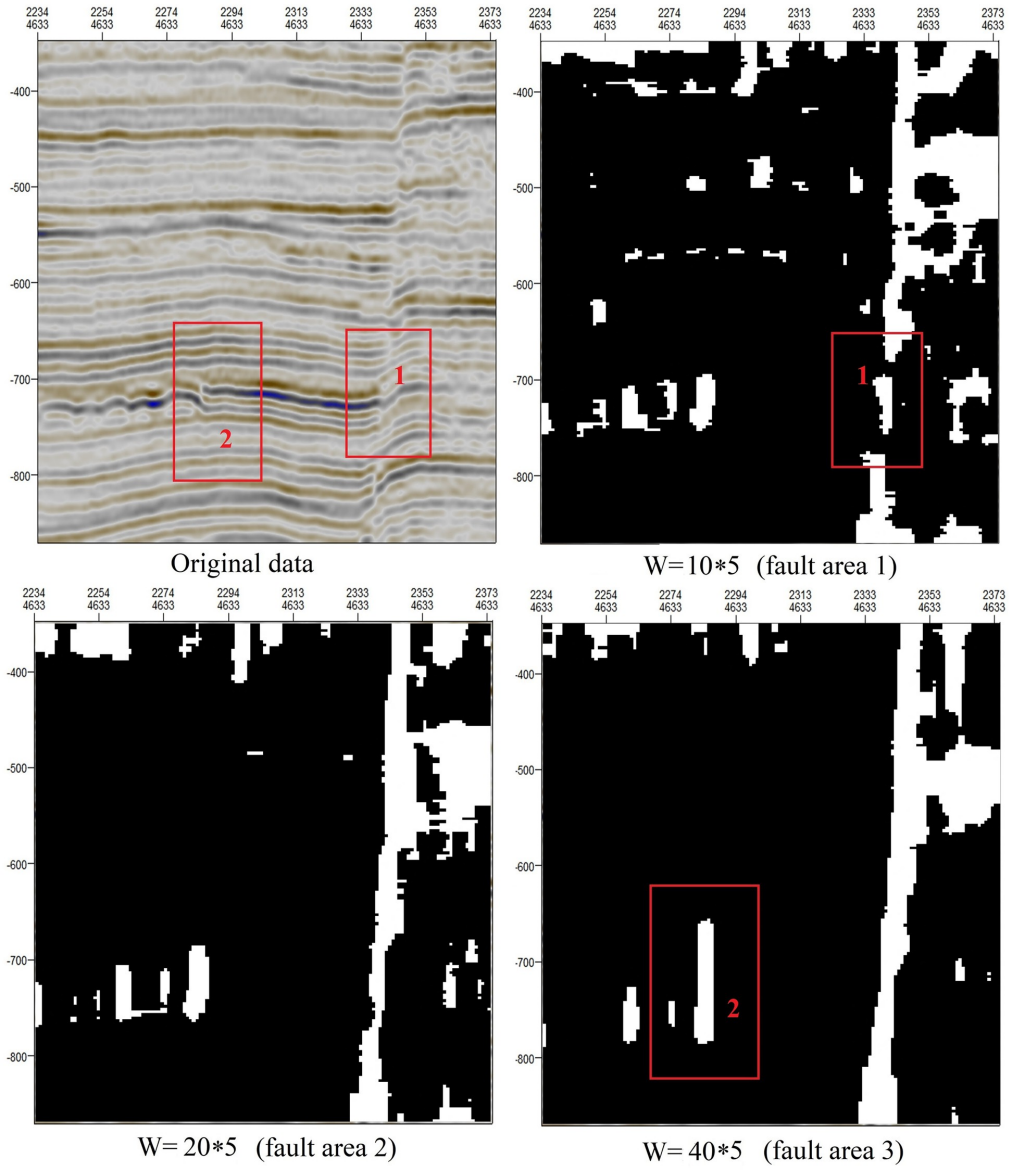
Influence of different values of m in the convolution kernel W on the extracted fault areas.

Fig 9 shows the effects of different values of m on fault extraction. We can draw the following conclusions: When m = 10, the fault continuity is poor (red-boxed region 1 in fault area 1). When m = 35, the longitudinal development characteristics of a fault may be incorrectly amplified (red-boxed region 2 in fault area 3). Through testing on many fault areas of different scales, we have found that the best range of m is between 15 and 30.

**Fig 10:**
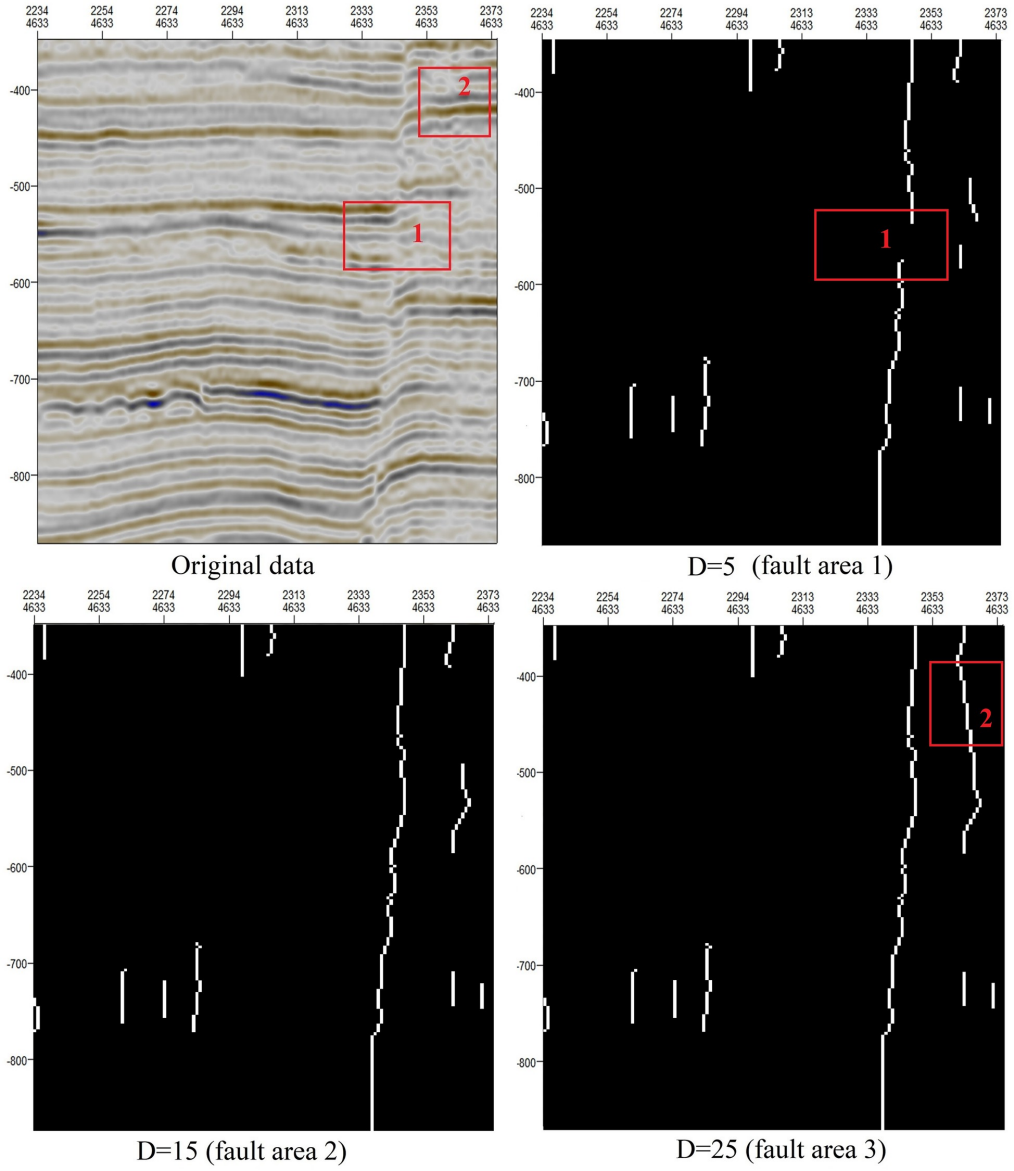
Influence of different values of D on fault continuity.

Connecting adjacent points can ensure the continuity of faults. Fig 10 shows the effect of the minimum distance considered when connecting adjacent points (D). We can draw the following conclusions: When D = 5, the continuity of the fault lines may be poor (red-boxed region 1 in fault area 1). When D = 25, erroneous fault lines may appear (red-boxed region 2 in fault area 3). Through testing on many fault areas of different scales, we have found that the best results are obtained when D is approximately 15.

**Fig 11:**
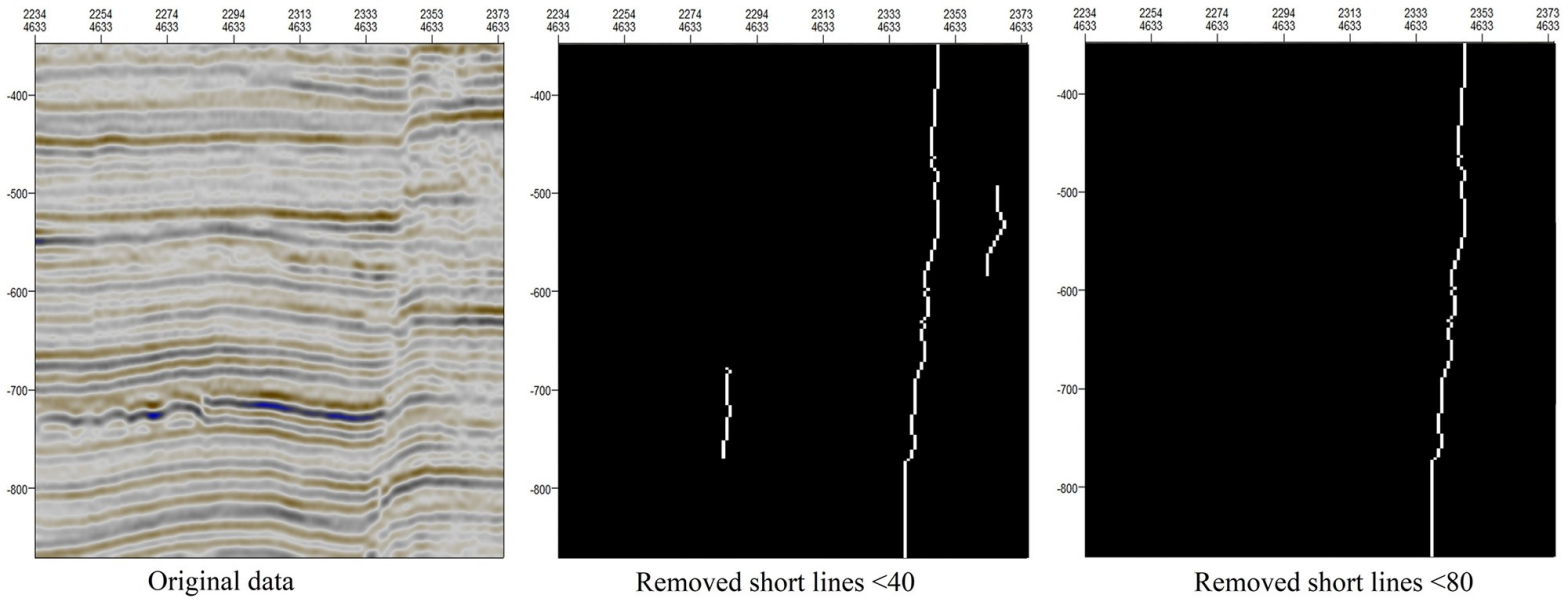
Influence of the removal of short lines of different lengths on the results of fault extraction.

The removal of short lines can allow faults of different scales to be identified. Fig 11 shows the results obtained when short lines of different lengths are removed. We can draw the following conclusions: When lines shorter than 40 are removed, some subtle faults or nonstructural factors are still identified as faults. When lines shorter than 80 are removed, only relatively large faults are identified. In this case, the size of area 1 in Fig 1 is 64*72; therefore, in area 1, the maximum length is less than 80, no faults will be detected.

Based on the above tests, we can draw the following conclusions:

1. The convolution kernel (W) used to extract the orientation field consistency is represented by m*n. When m is large, small faults will be enlarged, but small nonfault discontinuities may also be incorrectly enlarged (fault area 3 in Fig 9). When m is small, the longitudinal characteristics of faults are not obvious. When n is large, two adjacent distinct faults may be merged into one (fault area 3 in Fig 8). When n is small, locations where the stratum is bent but there is no fault may be incorrectly identified as faults (fault area 1 in Fig 8).
2. When the minimum distance D considered when connecting adjacent points is relatively small, the fault continuity is poor (fault area 1 in Fig 10), but fracture tracking may be more accurate. When D is large, the fault continuity is good, but errors in fracture tracking may occur (fault area 3 in Fig 10).
3. As shown in Fig 11, when the length of removed short lines is long, only large faults can be extracted. When the length of removed short lines is shorter, smaller faults may be extracted.
4. Taking the SEG-Y seismic data from Parihaka, New Zealand, as an example, when we need to identify large faults, the optimal parameters are a convolution kernel of 30*8, a minimum fault separation distance of 15, and a minimum fault length of 80. When we need to identify small faults, the continuity of large faults will be relatively poor, and the optimal parameters are a convolution kernel of 15*5, a minimum fault separation distance of 15, and a minimum fault length of 40.

### Comparison

**Fig 12:**
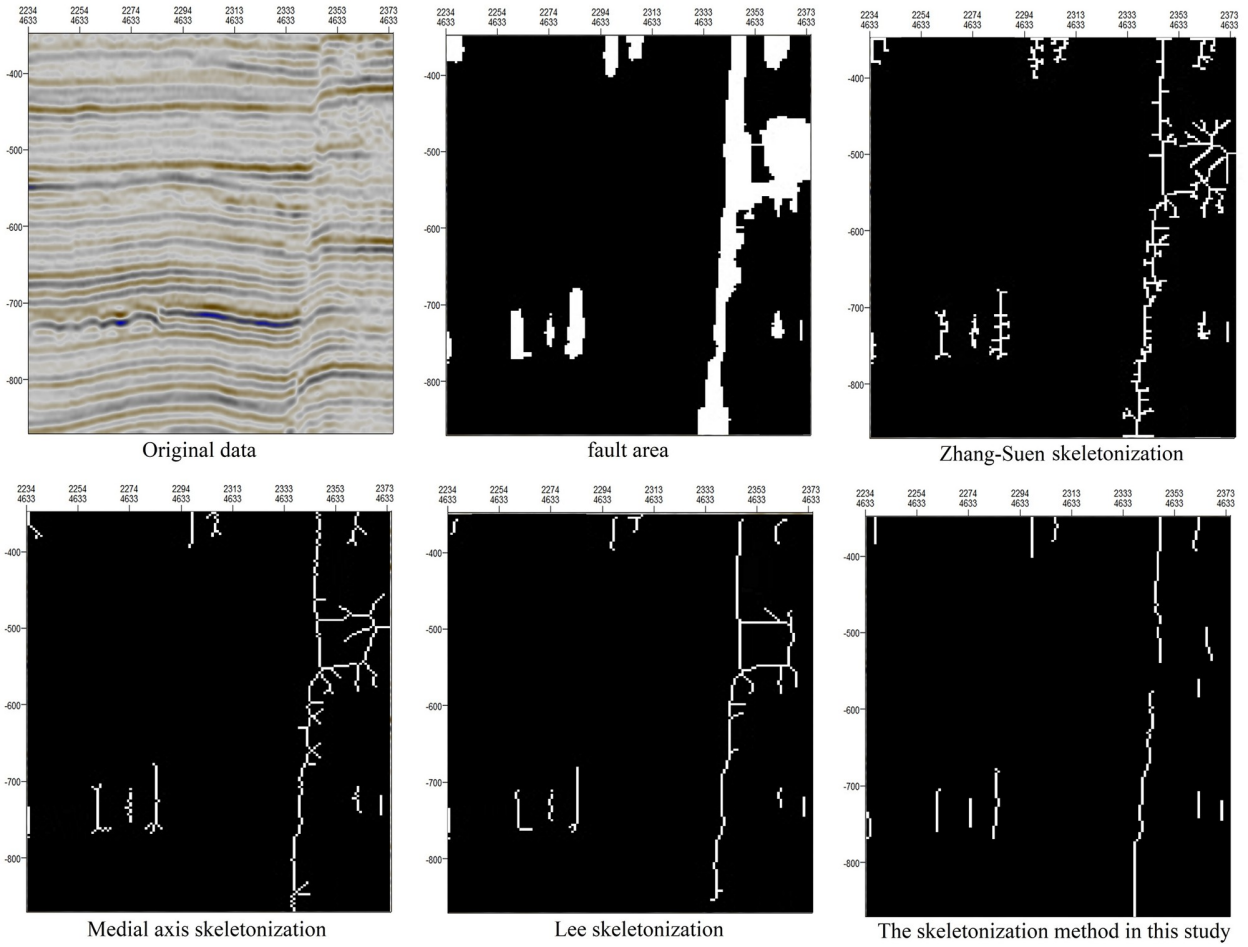
Comparison of different skeletonization methods.

There are many available skeleton extraction methods. To illustrate the advantages of the skeletonization method presented in this paper, the proposed method is compared with those of Zhang-Suen and Lee and with medial axis skeletonization. As shown in Fig 12, the skeletonization method presented in this paper preserves and enhances the obvious longitudinal development characteristics of faults and results in fewer burrs.

**Fig 13:**
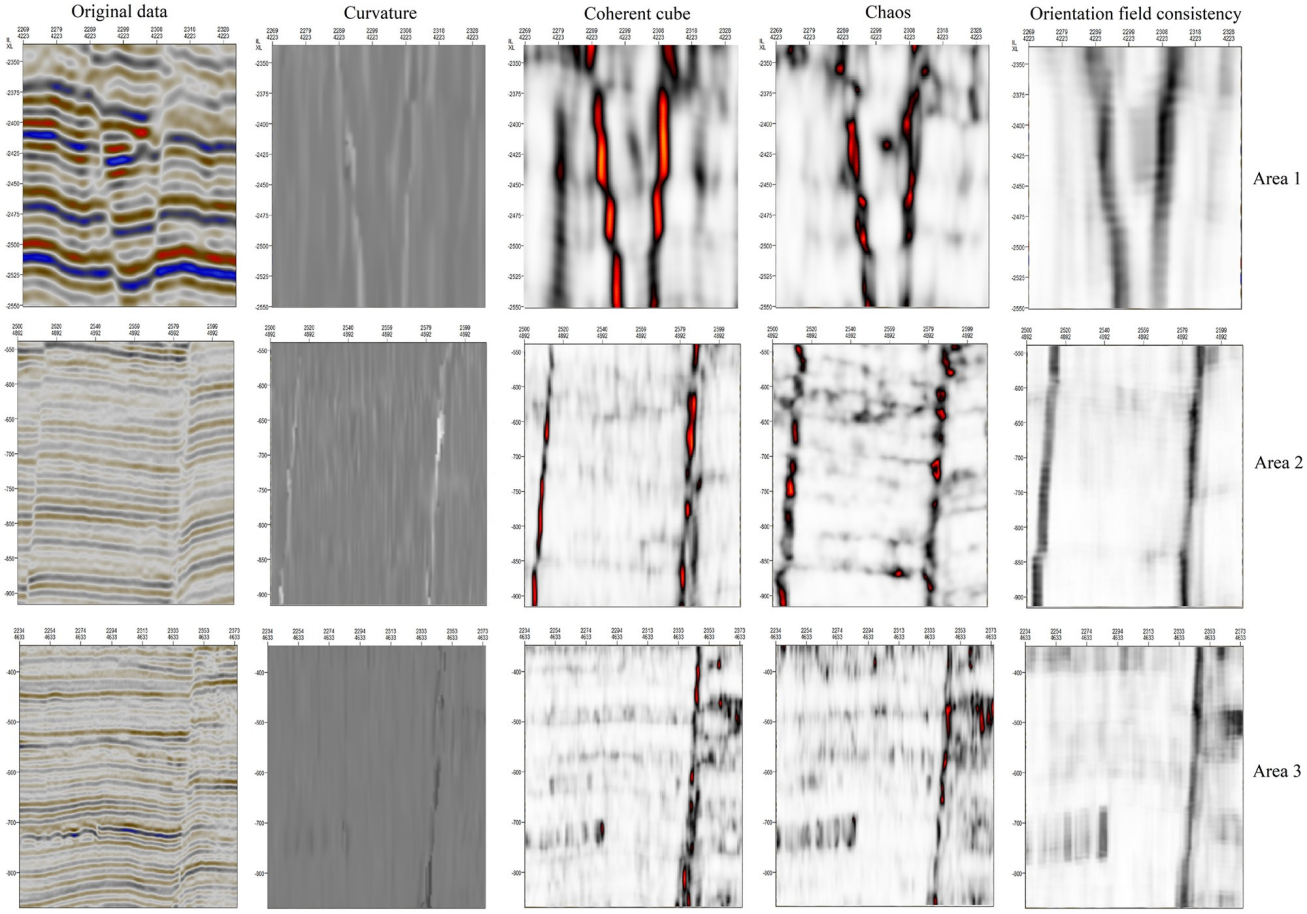
Comparison of different fault enhancement methods.

Commonly used fault enhancement approaches include the curvature, coherence cube, and chaos methods. The following conclusions can be drawn by comparing the fault enhancement results of our orientation field consistency method with those of these common methods (Fig 13):

1. The fault enhancement effects of coherence cube, chaos, and curvature extraction are similar to those of orientation field consistency extraction, indicating that the proposed method can be used in place of other methods.
2. Compared with that of the fault enhancement results based on the orientation field consistency, the contrast of curvature-based results is weak, necessitating further image processing to enhance the visual effect of fault display.
3. Compared with the orientation field consistency method, the coherence cube method of fault enhancement requires the adjustment of too many parameters (including the IL range, XL range, longitudinal smoothing, IL scale, XL scale, vertical scale, plane confidence threshold, and dip-guided smoothing) to obtain better results. In contrast, fault enhancement based on orientation field consistency requires adjusting only the convolution kernel.
4. Compared with that of the chaos method, the sensitivity of the orientation field consistency method of fault enhancement to transverse discontinuities is weaker.

**Fig 14:**
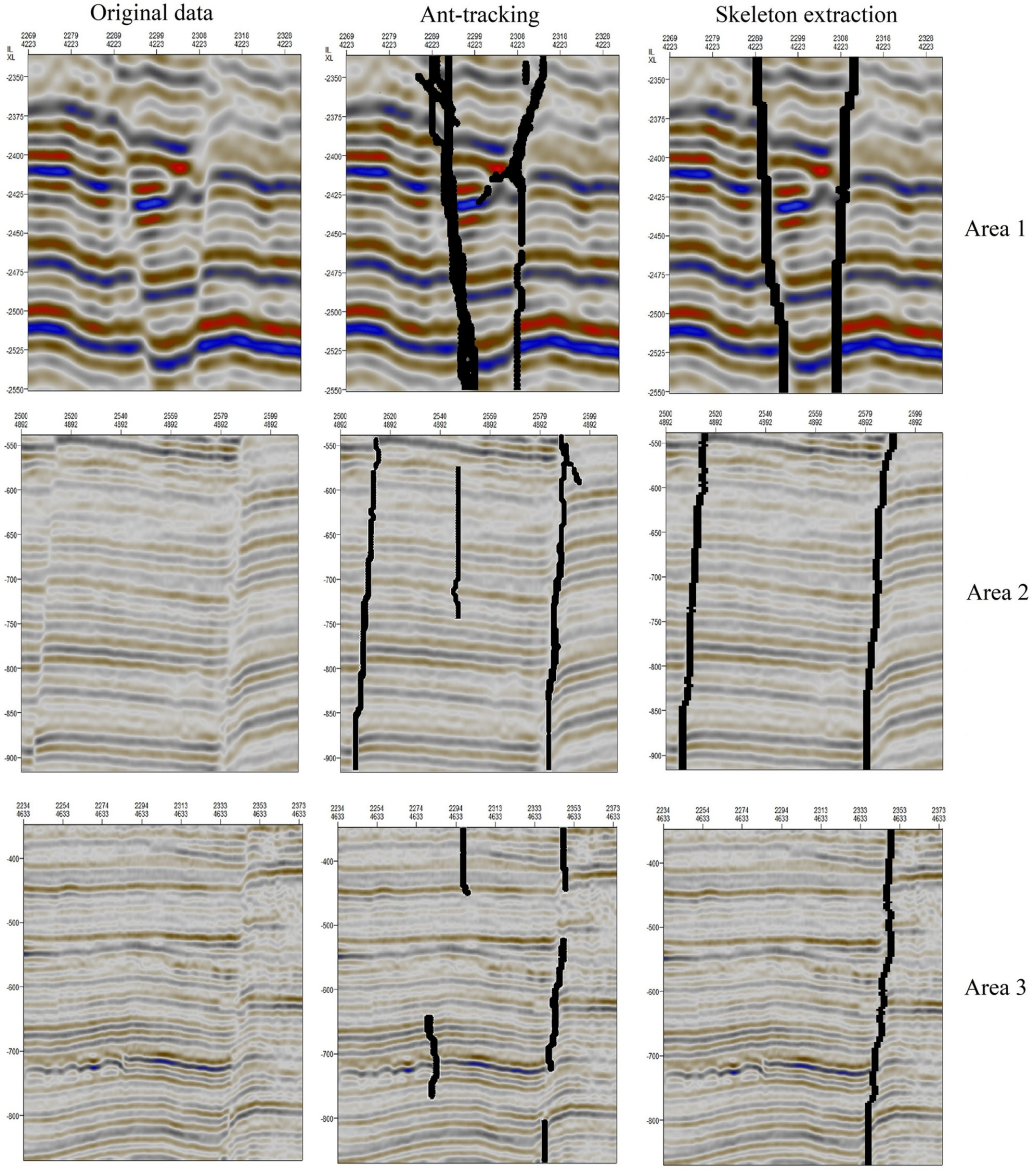
Comparison of ant tracking and skeleton extraction.

The traditional ant tracking method achieves high efficiency in tracking fault lines. This approach has been recognized by many scholars and has been widely adopted. Based on the orientation field consistency method of fault enhancement, the ant tracking method is compared with the skeleton extraction method proposed in this paper to prove the effectiveness of the latter. Table 1 shows the parameters to be adjusted for ant tracking and the method proposed in this paper. Fig 14 shows the fault detection effects for three seismic areas, where the black tracks are the faults extracted by the two algorithms. The following conclusions can be drawn:

1. Table 1 shows that the ant tracking parameters require excessive adjustment, and improper parameter adjustment will affect the accuracy of fault line recognition. In comparison, the method presented in this paper has fewer parameters to adjust, thereby simplifying the cumbersome parameter adjustment process.
2. In the ant tracking method, cluster analysis is performed in accordance with the number of pheromones; consequently, the continuity of this method is poor. In the method proposed in the present paper, the continuity of the fault lines is strengthened by the closing operation and the connection of adjacent points. Therefore, Fig 14 shows that the method proposed in this paper has an advantage in terms of fault continuity.
3. In ant tracking, the extraction of faults or fractures along the longitudinal direction is controlled by manually adjusting the parameters, and the results are sometimes sensitive to transverse discontinuities. In the method of this paper, the longitudinal center points are extracted from binary fault areas to control the longitudinal characteristics of the fault lines, and there is no need to adjust the parameters to control the longitudinal nature of the extracted fault lines. Therefore, the sensitivity of this method to transverse discontinuities is very low.

**Table 1:** Comparison of ant tracking and skeleton extraction parameters.

Ant tracking	Skeleton extraction
Initial ant boundary Ant track deviation Ant step size Illegal steps allowed Legal steps required Stopping criteria Azimuth Dip	Convolution kernel Minimum fault separation distance for the connection of adjacent points Minimum fault length for the removal of short lines

## Conclusions

A skeleton extraction method based on orientation field consistency is proposed for fault detection in this paper. The results of the proposed method are compared with those obtained using the ant tracking method. The following conclusions are drawn.

1. The fault enhancement results extracted on the basis of orientation field consistency have the following advantages. Compared with the curvature method, the proposed method achieves better contrast and a better visual effect. Compared with the coherence cube method, fewer parameter adjustments are required. Compared with the chaos method, transverse discontinuities are more effectively suppressed.
2. In principle, the ant tracking method depends on cluster analysis based on the number of pheromones, so the continuity of the extracted faults is weaker. In the method proposed in this paper, the continuity of the fault lines is strengthened by the closing operation and connection of adjacent points.
3. The ant tracking method requires the adjustment of too many parameters, and improper parameter adjustments can significantly affect the accuracy of fault line extraction. The method proposed in this paper requires the adjustment of fewer parameters, thereby simplifying the fault extraction process to a certain extent.
4. In ant tracking, the extraction of longitudinal faults is controlled through the manual adjustment of parameters. Sometimes, ant tracking is sensitive to lateral discontinuities. In the method of this paper, longitudinal center points are extracted from binarized fault areas to control the longitudinal characteristics of the fault lines, and there is no need for parameter adjustment to control longitudinal fault extraction. Therefore, the method proposed in this paper is advantageous in suppressing transverse discontinuities.
